# The impact of universal newborn hearing screening on long-term literacy outcomes: a prospective cohort study

**DOI:** 10.1136/archdischild-2014-307516

**Published:** 2014-11-26

**Authors:** Hannah Pimperton, Hazel Blythe, Jana Kreppner, Merle Mahon, Janet L Peacock, Jim Stevenson, Emmanouela Terlektsi, Sarah Worsfold, Ho Ming Yuen, Colin R Kennedy

**Affiliations:** 1 Faculty of Medicine, University of Southampton, Southampton, UK; 2 Faculty of Social and Human Sciences, University of Southampton, Southampton, UK; 3 Developmental Science Research Department, UCL, London, UK; 4 Division of Health and Social Care Research, King's College London, NIHR Biomedical Research Centre at Guy's and St Thomas’ NHS Foundation Trust and King's College London, London, UK; 5 University of Southampton and University Hospital Southampton NHS Foundation Trust, Southampton, UK

**Keywords:** Screening, Deafness, Outcomes research, Neurodevelopment

## Abstract

**Objective:**

To determine whether the benefits of universal newborn hearing screening (UNHS) seen at age 8 years persist through the second decade.

**Design:**

Prospective cohort study of a population sample of children with permanent childhood hearing impairment (PCHI) followed up for 17 years since birth in periods with (or without) UNHS.

**Setting:**

Birth cohort of 100 000 in southern England.

**Participants:**

114 teenagers aged 13–19 years, 76 with PCHI and 38 with normal hearing. All had previously their reading assessed aged 6–10 years.

**Interventions:**

Birth in periods with and without UNHS; confirmation of PCHI before and after age 9 months.

**Main outcome measure:**

Reading comprehension ability. Regression modelling took account of severity of hearing loss, non-verbal ability, maternal education and main language.

**Results:**

Confirmation of PCHI by age 9 months was associated with significantly higher mean z-scores for reading comprehension (adjusted mean difference 1.17, 95% CI 0.36 to 1.97) although birth during periods with UNHS was not (adjusted mean difference 0.15, 95% CI −0.75 to 1.06). The gap between the reading comprehension z-scores of teenagers with early compared with late confirmed PCHI had widened at an adjusted mean rate of 0.06 per year (95% CI −0.02 to 0.13) during the 9.2-year mean interval since the previous assessment.

**Conclusions:**

The benefit to reading comprehension of confirmation of PCHI by age 9 months increases during the teenage years. This strengthens the case for UNHS programmes that lead to early confirmation of permanent hearing loss.

**Trial registration number:**

ISRCTN03307358.

**What is already known on this topic**

- Universal newborn hearing screening (UNHS) is an effective way of increasing rates of early identification of congenital permanent childhood hearing impairment (PCHI).
- UNHS and early identification of PCHI are associated with benefits to language and reading outcomes in middle childhood.

**What this study adds**

- This study is the first to describe the effects of UNHS and early confirmation of PCHI on longer-term literacy outcomes.
- Early confirmation of PCHI was associated with significant benefits to reading comprehension in the teenage years.
- The benefit of early confirmation of PCHI to reading comprehension had increased from moderate to large between the ages of 8 and 17 years.

## Introduction

Bilateral permanent childhood hearing impairment (PCHI) of moderate, severe or profound severity is the commonest sensory disability affecting 1 in 750 children and is present at birth in more than 80% of affected children.^1^ PCHI of this degree has a detrimental impact on all aspects of oral language development^2–5^ and impacts significantly on skills that depend on language ability, such as reading and writing.^6^ ^7^

Identification of PCHI in early childhood enables affected children to receive early intervention to optimise their language access during a ‘sensitive period’ for language development.^8^ More than half of babies born with PCHI do not have prospectively identifiable risk factors so that only universal newborn hearing screening (UNHS) programmes can identify the majority of those affected. UNHS, when first introduced in the UK, more than doubled the proportion confirmed by 9 months to three-quarters of all cases of bilateral PCHI >40 dB.^9^ ^10^ We have previously reported that children with PCHI from that birth cohort had significant benefits to language and reading at age 6–10 years associated with birth in periods with UNHS and with confirmation of PCHI by age 9 months.^11–13^

Systematic reviews have been increasingly supportive of UNHS^14–16^ and both the UK National Screening Committee and the US Preventative Services Task Force have recommended in favour of it.^17–19^ During the calendar year of 2009, an estimated 5073 cases of PCHI were detected by UNHS in the USA, accounting for 43% of all detected cases of the 29 medical conditions for which newborn screening is recommended.^20^

Both the US Preventative Services Task Force^15^ ^17^ and a 2009 WHO report on UNHS^21^ have, however, drawn attention to the evidence gap regarding benefits beyond primary school age and benefits to functional outcomes. This study consequently aimed to provide novel evidence regarding the effects of UNHS and early confirmation of PCHI on functional outcomes in the teenage years. We report findings regarding the abilities of teenagers with PCHI at age 17 years whom we previously assessed at age 8 years.^11^ ^12^ Reading is a skill that is dependent on underlying language ability^22^ ^23^ that relates very closely to educational and employment outcomes, and as such is a key functional outcome.^24^ Reading comprehension was therefore prespecified as the primary outcome in this study.

## Patients and methods

**Figure 1:**
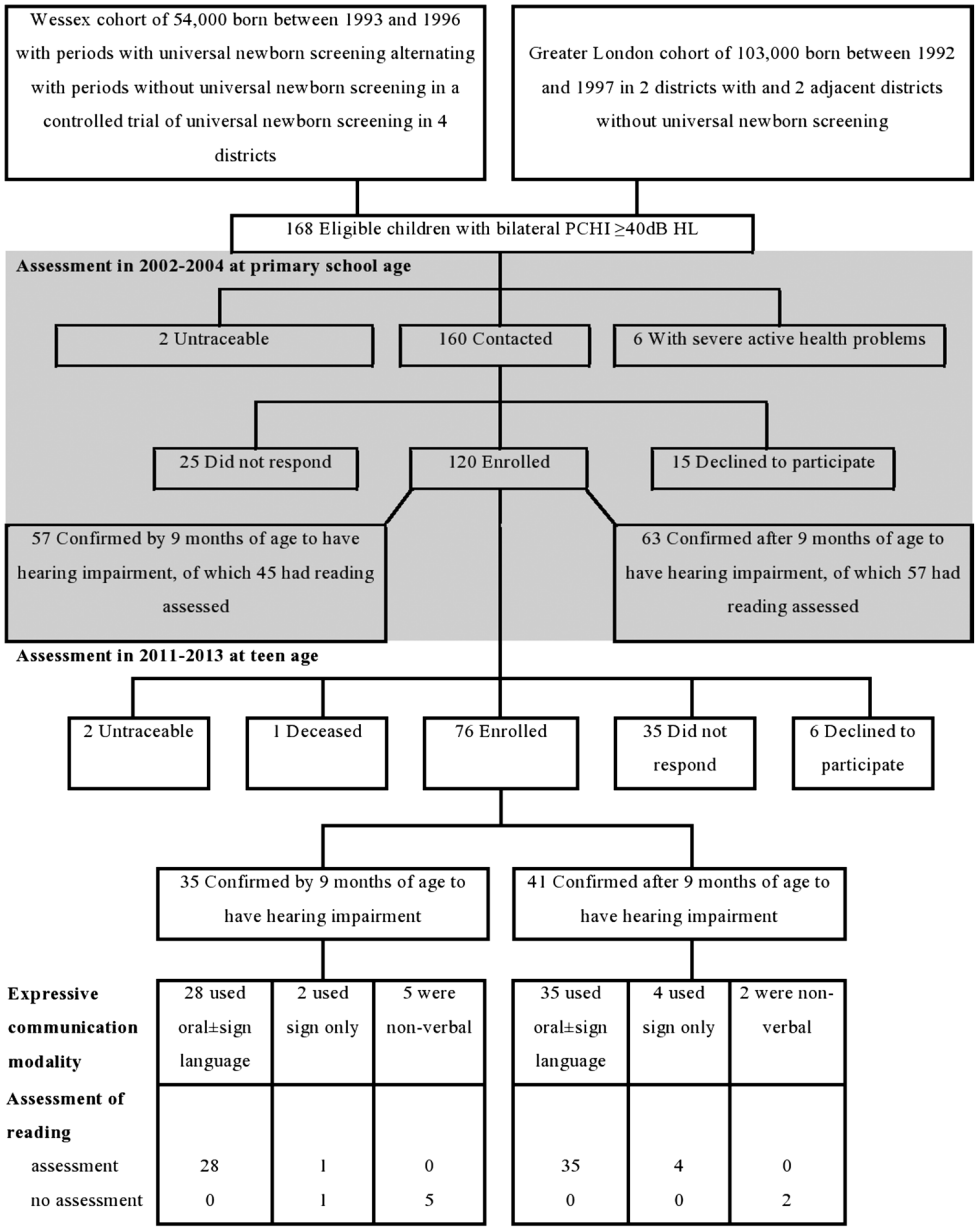
Numbers of teenagers with permanent childhood hearing impairment who were eligible for the study and assessed for reading ability at primary school and teenage. Greyed out section of the figure indicates the previous study at age 6–10 years. dB HL, decibels hearing level.

The children in this prospective follow-up study, 120 children with bilateral PCHI >40 decibels hearing level (dB HL) (not known to be postnatally acquired) and a comparison group of 63 normally hearing children, were drawn from a birth cohort of 157 000 children born in eight districts of southern England (see online supplementary appendix 1), of whom about half were born in periods with UNHS. We previously reported a number of details relating to this population in infancy and first decade, including the UNHS programmes for PCHI to which they were exposed; the service provision by district and regional audiology and by other services for confirmation and management of their PCHI; and the language and reading abilities of the children at 6–10 years.^9–13^ ^25–31^ Nine years after their previous language and reading assessments at 6–10 years, 76 (63%) teenagers with PCHI and 38 (60%) of the normally hearing comparison group have now participated in the study we report here (figure 1). We estimate that 49% of all oral language users with PCHI from the birth cohort had their reading assessed at age 17.1 years (see online supplementary appendix 1).

### Procedure

Each participant was assessed by a trained researcher, unaware of their audiological history, using the York Assessment of Reading for Comprehension Secondary Edition,^32^ a standardised reading test that provides measures of accuracy, comprehension and summarisation skill (see online supplementary appendix 1). A 20 min timed version^33^ of Raven's Standard Progressive Matrices Plus^34^ was used as a measure of non-verbal ability. The preplanned primary outcome of our study was reading comprehension score after adjustment in a multiple linear regression for severity of hearing loss, non-verbal ability and maternal education, which were recognised as potential confounders of the primary outcome.^11^ Adjusted reading accuracy and reading summarisation ability z-scores were preplanned secondary outcomes.

Severity of hearing impairment was categorised from the most recent audiological evaluation at audiology and cochlear implant clinics as moderate (40–69 dB HL), severe (70–94 dB HL) or profound (≥95 dB HL) according to four-frequency averaging of the pure-tone thresholds at 0.5, 1, 2 and 4 kHz. Maternal education was classified according to the 2001 census in the UK.

This study was approved by the Southampton and SW Hampshire Research Ethics Committee. Written informed consent for participation in the study was obtained from principal caregivers and from the teenage participants themselves.

### Analysis strategies

The primary outcome (reading comprehension) and the analysis strategy were prespecified and the statistical analysis plan was written before examination of the data. The target sample size of 96 with half of the sample born in periods with (or without) UNHS, or, in a parallel set of analyses, exposed to early (or late) confirmation of PCHI, was sufficient to have 90% power to detect a standardised difference in the primary outcome of at least 0.67 SDs at a 5% significance level (two sided) using a uni-factorial test. We prespecified the definition of ‘early’ confirmation of PCHI as confirmation by nine completed months of age, consistent with the definition used in our previous trial of UNHS^9^ and with the US Preventive Services Task Force benchmark for diagnosing and treating infants before 10 months of age.^15^ ^16^

The group mean and SD reading scores in the normally hearing comparison group were used to derive z-scores for the teenagers with PCHI where the mean and SD in the normally hearing group was 0 and 1, respectively. The z-scores in the participants with PCHI were thus expressed in terms of the number of SDs from the mean in the normally hearing comparison group. Analyses were run both with and without British Sign Language users. This did not alter the pattern of results which are therefore presented for the combined group of oral and signing communicators. Where statistically significant inter-group differences were found, subgroup analysis was then undertaken in those who had and had not received cochlear implants. The method of adjusting reading z-scores appropriately to look at change in reading ability over time comparing current scores with those previously obtained at aged 6–10 years is described in online supplementary appendix 1.

We assessed in a linear regression model the relationships between birth during periods of UNHS or confirmation of PCHI by age 9 months and age-adjusted reading z-scores (using Stata/SE V.12.1) in oral and signing communicators (see online supplementary appendix 1). The extent to which the effect of early confirmation made a significant additional contribution to model fit after screening was included in the model was tested with a likelihood ratio test. Normality and homogeneity of the residual variance were examined for all measures to ensure that the regression models were appropriate.

## Results

The 114 participating teenagers were similar to the 183 who had previously participated in the study of reading and language at 7.9 years with regard to sex, non-verbal ability and maternal educational level at the time of the previous study (table 1). The 76 participants with PCHI (figure 1) were similar to the 120 who had previously participated with regard to severity of PCHI, exposure to UNHS and confirmation of PCHI prior to nine completed months from birth (table 1). These characteristics were also similar between those who had their PCHI confirmed by age 9 months (n=35) and those who had it confirmed later (n=41) (table 2) and between those who were born in periods with UNHS (n=37) and those who were not (n=39) (data not shown). The early and late confirmed PCHI groups were similar with respect to the percentages affected by cerebral palsy, visual disability or learning disability (table 2). These groups were also similar in that the aetiology of deafness was identified in about half and was genetic in 34%–43% (table 2). The proportion of teenagers in whom English was not the main language at home was higher in the late (12%) than the early (3%) confirmed group and adjustment for this variable was therefore included in the regression model in addition to the three prespecified variables (see Patients and methods section).

**Table 1:** Demographic characteristics of participants and non-participants in the current study of reading ability in teenagers

	Children with bilateral PCHI		Normally hearing children	
Characteristic	Whole sample* (n=120)	Teenage sample participating in present study (n=76)	Whole sample* (n=63)	Teenage sample participating in present study (n=38)
Mean age (SD) (range) in years At primary school assessment	7.9 (1.3) (5.4 to 11.7)	7.9 (1.1) (5.8 to 10.7)	8.1 (1.0) (6.2 to 9.8)	8.0 (1.1) (6.2 to 9.8)
Female sex n (%)	53 (44)	37 (49)	26 (41)	13 (34)
Severity of hearing loss n (%)				
Moderate	62 (52)	38 (50)	NA	NA
Severe	29 (24)	16 (21)		
Profound	29 (24)	22 (29)		
Born in periods with UNHS n (%)	61 (51)	37 (49)	NA	NA
PCHI confirmed ≤9 months n (%)	57 (48)	35 (46)	NA	NA
English as main language at home n (%)	99 (83)	67 (88)	60 (95)	36 (95)
Maternal education n (%)				
No qualifications or <5 O-levels†	43 (36)	24 (32)	25 (40)	11 (29)
≥5 O-levels or some A-levels†	62 (52)	40 (53)	25 (40)	16 (42)
University or higher degree	14 (12)	12 (16)	13 (21)	11 (29)

*The ‘whole sample’ was a population-based sample of children with PCHI and a normally hearing comparison group that participated 9 years earlier in a study of language and reading at primary school age.

†O-level examinations (now replaced by general certificates of education) are usually taken at 16 years of age; A-level examinations (now replaced by A2s) are taken 2 years later as qualifications for entry to higher education.

NA, not applicable; PCHI, permanent childhood hearing impairment (see Patients and methods section for detailed definition of degree of PCHI); UNHS, universal newborn hearing screening.

**Table 2:** Characteristics of participating teenagers with hearing impairment and with normal hearing

	Children with bilateral PCHI (n=76)		Normally hearing children (*n*=38)
Characteristic	Confirmation of PCHI at ≤9 months (n=35)	Confirmation of PCHI at >9 months (n=41)	
Mean (SD) age at assessment in years	16.8 (1.5)	17.3 (1.3)	16.3 (1.2)
Female sex n (%)	16 (46)	21 (51)	13 (34)
Born in period with UNHS n (%)	23 (66)	14 (34)	NA
Severity n (%)			
Moderate*	16 (45)	17 (41)	
Severe	7 (20)	12 (29)	NA
Profound	12 (34)	12 (29)	
Hearing device used n (%)			
Cochlear implant/s	7 (20)	8 (19)	
Hearing aid/s	23 (66)	32 (78)	NA
No hearing device	5 (14)†	1 (2)‡	
Mean (SD) non-verbal ability z-score§	−0.3 (0.9)	−0.3 (0.8)	0 (1)
Aetiology n (%)			
Syndromic	9 (26)	4 (10)	
Other hereditary	6 (17)	10 (24)	NA
Known non-genetic risk¶	2 (6)	3 (7)	
Not known	18 (51)	24 (59)	
Other disabilities n (%)			
Cerebral palsy	1 (3)	1 (2)	0
Visual disability	1 (3)	1 (2)	0
Learning disability	6 (17)	8 (20)	0
None of the above	28 (80)	33 (80)	38 (100)
English as main language at home n (%)	34 (97)	36 (88)	36 (95)
Maternal education n (%)			
No qualifications/<5 O-levels**	9 (26)	10 (24)	6 (16)
≥5 O-levels or some A-levels**	17 (49)	21 (51)	14 (37)
University or higher degree	9 (26)	10 (24)	18 (47)

*Six participants (two with confirmation of PCHI at ≤9 months, four with confirmation of PCHI >9 months) classified with PCHI of moderate severity when previously assessed at 6–10 years of age had shown improvements by the current study such that their better ear hearing thresholds now fell between 30 and 40 dB.

†Three with significant additional impairments (all had chromosomal disorders and learning disability), two with moderate PCHI who were not current hearing aid users.

‡One with significant additional impairments (learning disability).

§Age-adjusted z-scores are listed for Ravens Progressive Matrices total score. The z-scores are the number of SDs of the scores in normally hearing children by which the age-adjusted score differed from the mean score in the normally hearing children.

¶Prematurity or cerebral palsy.

**O-level examinations (now replaced by general certificates of education) are usually taken at 16 years of age; A-level examinations (now replaced by A2s) are taken 2 years later as qualifications for entry to higher education.

NA, not applicable; PCHI, permanent childhood hearing impairment; UNHS, universal newborn hearing screening

The early and late confirmed groups showed mean reading comprehension z-scores that were 0.63 and 1.74 SDs, respectively, below the mean reading z-score in the normally hearing comparison group (table 3). The teenagers who had their hearing impairment confirmed by nine completed months of age had significantly higher adjusted mean z-scores than the later confirmed teenagers for both reading comprehension (1.17 SD) and reading summarisation (0.96 SD) (table 3). These effect sizes were larger in the 78% (51/65) who had not received cochlear implants (adjusted inter-group differences 1.29, 95% CI 0.52 to 2.07, p=0.002; 1.00, 95% CI 0.30 to 1.70, p=0.006, respectively). Adjusted inter-group z-score differences on the three reading outcome measures between all teenage participants who were or were not born in periods with UNHS at birth were smaller (0.09 to 0.22) and not statistically significant (table 3).

**Table 3:** Reading z-scores for children with bilateral PCHI by age of confirmation of PCHI and by birth in periods with and without UNHS

Measure	Number of observations		Mean z-score (SD)		Unadjusted mean difference (95% CI) a–b	p Value	Adjusted* mean difference (95% CI) a–b	p Value
	PCHI confirmed at ≤9 months (n_1_)	PCHI confirmed at >9 months (n_2_)	PCHI confirmed at ≤9 months (a)	PCHI confirmed at >9 months (b)				
YARC reading comprehension	28	37	−0.63 (1.63)	−1.74 (1.50)	1.11 (0.33 to 1.89)	0.006	1.17 (0.36 to 1.97)	0.005
YARC reading summarisation	28	37	−0.56 (1.37)	−1.36 (1.44)	0.80 (0.09 to 1.51)	0.03	0.96 (0.24 to 1.68)	0.01
YARC reading accuracy	27	33	−1.12 (1.69)	−1.71 (1.44)	0.59 (−0.22 to 1.40)	0.15	0.68 (−0.09 to 1.46)	0.08

	UNHS (n_1_)	No UNHS (n_2_)	UNHS (a)	No UNHS (b)	a—b		a—b	

YARC reading comprehension	33	32	−1.15 (1.87)	−1.37 (1.39)	0.22 (−0.60 to 1.04)	0.60	0.15 (−0.75 to 1.06)	0.73
YARC reading summarisation	33	32	−0.97 (1.51)	−1.07 (1.43)	0.10 (−0.62 to 0.83)	0.78	0.22 (−0.58 to 1.03)	0.58
YARC reading accuracy	31	29	−1.43 (1.59)	−1.47 (1.58)	0.04 (−0.78 to 0.86)	0.93	0.09 (−0.76 to 0.93)	0.84

*Adjusted for severity of PCHI, maternal education level, non-verbal ability and English as main language at home.

PCHI, permanent childhood hearing impairment; UNHS, universal newborn hearing screening; YARC, York Assessment of Reading for Comprehension.

Change in the estimates of effect sizes and p values of early confirmation and of screening was minimal when they were modelled together rather than separately, suggesting that these effects were working independently (see online supplementary table e1). Adding the effect of early confirmation into the regression model after screening was included made a significant additional contribution to model fit (likelihood ratio test χ^2^=7.61, p=0.006) indicating that early confirmation of PCHI accounted for significant unique variance in reading outcomes beyond that accounted for by exposure to UNHS.

**Figure 2:**
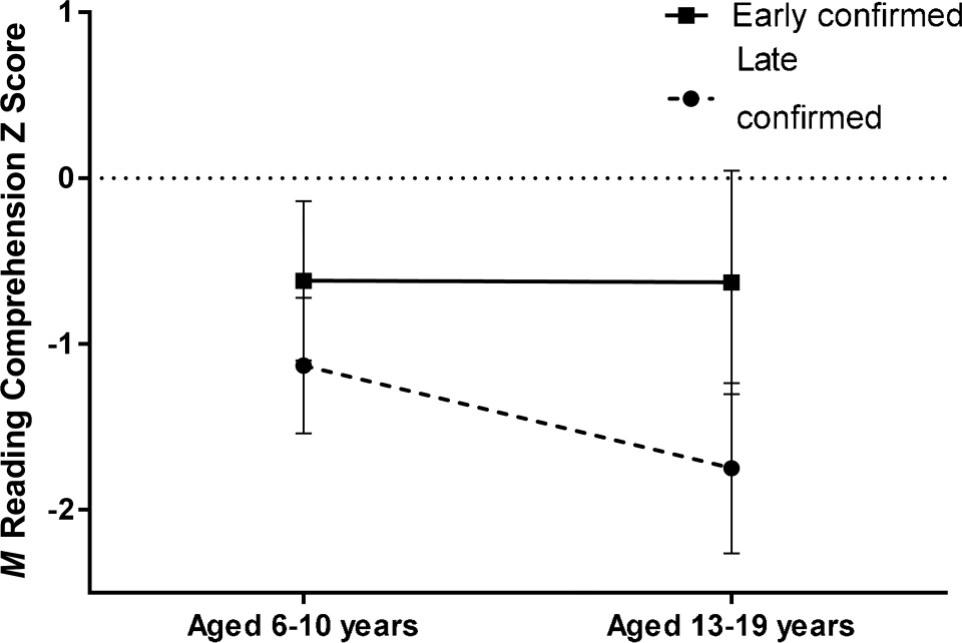
Unadjusted mean reading comprehension z-scores in children with permanent childhood hearing impairment at age 6–10 years and 13–19 years by age of confirmation of hearing impairment. Error bars represent the 95% CI of the means in the 26 early confirmed and 36 late confirmed participants who provided data at both primary school age and teenage.

Comparison of the recalculated reading comprehension z-scores at primary school age (see Patients and methods) with those at age 13–19 years from the present study showed that unadjusted reading comprehension z-scores remained nearly unchanged in the early confirmed group but decreased (ie, became more negative relative to the hearing control group) in the late confirmed group (figure 2). Compared with that in the late confirmed group, the adjusted mean annual rate of change in the reading comprehension z-score during the 9.2 year interval between primary school and teenage assessments was less negative in the early confirmed group (mean inter-group rate difference 0.06 per year, 95% CI −0.02 to 0.13, p=0.14). This inter-group difference in annual rate of change of reading comprehension was larger and statistically significant in those who had not received cochlear implants (mean inter-group difference 0.08 per year, 95% CI 0.01 to 0.15, p=0.03).

## Discussion

This study of teenagers with PCHI who were involved in trials of UNHS is the first to describe the effects of UNHS and early confirmation of PCHI on outcomes beyond the primary school years. Confirmation of PCHI by nine completed months of age was associated with significantly better performance on reading comprehension, the prespecified primary outcome variable. Comparing the scores from the current study with reading scores obtained 9 years earlier from the same individuals, the teenagers whose PCHI had been confirmed early had maintained their level of performance relative to the hearing controls whereas the late confirmed teenagers had not: the gap between the early and late confirmed groups had doubled between the two assessments. The early superiority in the reading skills of the early confirmed participants may have enabled them to read more demanding reading material more frequently than their peers with later confirmed PCHI, thus increasing the skill gap between the two groups. The superiority resulting from this rich-get-richer ‘Matthew effect’^35^ was more than 1 SD of the range of reading comprehension scores in their normally hearing peers and is likely to impact on their life chances through educational achievement and employment.^24^

Non-verbal ability was very similar in the early and late confirmed groups and adjustment for it was included in the regression model. This suggests that the deficit in reading scores in the late confirmed participants did not result from a general cognitive deficit but rather from the specific impact of delayed access to optimal language input early in life on language-related abilities. The early and late confirmed groups did not show different proportions of genetic and non-genetic aetiologies of deafness nor of disabilities additional to deafness that might account for the observed differences in reading z-scores.

Factors other than age at confirmation of PCHI appeared to determine reading outcomes for that minority of participants who had received cochlear implants^36^ although this subgroup analysis was not preplanned and should be treated with caution. A greater dependency of teenage reading ability of the implanted subgroup on age at implantation than on age at confirmation may explain this difference but studies of larger numbers of cochlear implantees are needed to determine this.

The effects of early confirmation were seen in those born in periods with and without UNHS and the effect of UNHS appears to be dependent on the increase in rates of early confirmation of PCHI to which it leads. The same NHS district and regional audiology teams delivered, in almost all cases, the care of both screened and unscreened and of both early and late confirmed populations in this study^31^ and the different outcomes between these groups are likely to reflect the effect of UNHS and of early confirmation rather than any differences in the services to which they were exposed. A 2013 birth cohort in the UK would, nevertheless, be likely to show a much stronger relationship between birth in periods with UNHS and reading outcomes. Effective postscreening audiology and other services for those screening positive for PCHI in the newborn period, which were largely absent in the period from 1992 to 1997 for the population described in this report, are now in place^18^ ^19^ and therefore screening positive on UNHS in the UK would be more likely to lead to confirmation of PCHI by age 9 months.

The annual attrition rate (ie, 3% over 17 years since UNHS or 4% over the 9 years since assessment at primary school) among children with PCHI eligible for the present study is low for a teenage population with a chronic medical condition but limited the power of the study to examine change in reading comprehension between the primary school and teenage assessments. In spite of this limitation in power, the inter-group differences on the prespecified primary outcome of reading comprehension were large enough to be both statistically significant and clinically important.

## Conclusions

As the Millenium Development Goals project approaches its 2015 target, UNESCO, UNICEF, the World Bank and WHO are increasingly considering early child development, in which infant hearing is a critical component, as a key determinant of subsequent health^21^ ^37^ and this report is therefore timely. Confirmation of PCHI by nine completed months of age was associated with significantly better performance on reading comprehension, the prespecified primary outcome variable, and the effect size of this benefit of early confirmation of PCHI had increased from moderate to large between assessments at the ages of 8 and 17 years. This strengthens the case for national governments to fund UNHS programmes that increase the rates of early confirmation of PCHI in the many developed and developing countries where UNHS for PCHI is currently under discussion but not yet adopted as national policy.^38–40^

## Supplementary Material

Web supplement
