# Supplementary material for: The impact of universal newborn hearing screening on long-term literacy outcomes: a prospective cohort study
Source: Arch Dis Child. 2014 Nov 26;101(1):9–15. doi: 10.1136/archdischild-2014-307516 (PMC4717422; doi:10.1136/archdischild-2014-307516)
Supplement: Web supplement [file archdischild-2014-307516-s1.pdf]

Appendix 1

Birth Cohort to which this study relates

The birth cohort comprised two sub-cohorts: First, the 1993-1996 Wessex birth cohort of 54,000 babies enrolled in the Wessex trial in which a programme of UNHS was or was not in place in each pair of districts for birth cohorts born in alternate four- or six-month periods.[9,10] Second, the 1992-1997 Greater London birth cohort of 100,000 babies, born in four districts in Greater London, of which two were the only two districts in the UK (Waltham Forest; Hillingdon) that provided UNHS for PCHI in the early 1990s,[25,26] and the other two were districts geographically adjacent to them (Redbridge; Brent & Harrow, respectively).

Procedure for deriving Reading Ability Scores from the York Assessment of Reading Comprehension

Reading accuracy raw scores reflecting the total number of correct items out of a possible 70 were calculated from the single word reading test and used to select the two passages for reading at the appropriate difficulty level. The reading comprehension raw score reflected the average of the total number of comprehension questions answered correctly for these two passages of text, read silently. After the comprehension questions, the participants were asked, after the text had been removed, to summarise its main points thus providing the basis of the summarisation score. Tables provided in the test manual were used to convert the reading comprehension and summarisation raw scores to ability scores.

The five participants whose primary expressive communication mode was British Sign Language (BSL) signed the meaning of each word that they were able to read and therefore did not produce reading accuracy scores. They then completed the passage reading test in the same way as the oral language users, except that they worked with a researcher who communicated with them using BSL and they read, rather than listened to, the comprehension questions. They also signed, rather than spoke, their responses, which were transcribed directly by the researcher.

Estimated losses (attrition) of eligible children with PCHI from the studied birth cohort.

Of the 160 eligible and contactable children with PCHI in the birth cohort (Figure 1), 120 and 76 participated in the primary school (mean age 7.9 years) and current (mean age 17.1 years) studies respectively. Of these participants, 102 (85%) and 68 (89%) completed sufficient reading and cognitive assessment to be included in the respective reading analyses (Figure 1). In both cases these analysed groups included 100% of participating oral language users, numbering 97 and 63 in the respective studies. If the 40 non-participants at primary school age (Figure 1) were similar to the 120 participants, in that 81% of them were oral language users, those with PCHI eligible for that study would have contained 129 (=97+32) oral language users. Since 63 of these oral language users had assessments of language at a mean age of 17·1 years in the present study, the retention rate of oral language users over 17 years was estimated to be 63/129 or 49%.

Oral and signing communicators and other subgroup analyses

Longitudinal analysis

For examination of change between primary school and teenage assessments, the z-scores of participants with PCHI when aged 6-10 years were recalculated relative to the z-scores of the subset of the normally hearing comparison group that had also provided reading comprehension data in the current study. Thus the participants with PCHI were being compared to the same reference group of hearing participants at both time points. The adjusted annual rate of change in performance between the primary school and the present assessments was calculated for each participant by dividing the change in the recalculated z-score by the time interval between assessments for that individual and adjusting in the multiple linear regression model described above.

eTable 1. Joint effects of universal newborn hearing screening and of early confirmation on adjusted reading z-scores in children with bilateral permanent childhood hearing impairment

Measure	Adjusted[1] difference in mean z-score	95% CI	*p*
**YARC Reading Comprehension (N=65)**			
Early vs late confirmation of PCHI	1.23	0.38 to 2.07	0.005
UNHS (yes vs no)	0.22	-0.66 to 1.11	0.62
**YARC Reading Summarisation (N=65)**			
Early vs late confirmation of PCHI	0.98	0.22 to 1.74	0.012
UNHS (yes vs no)	0.08	-0.72 to 0.88	0.85
**YARC Reading Accuracy (N=60)**			
Early vs late confirmation of PCHI	0.71	-0.10 to 1.52	0.085
UNHS (yes vs no)PCHI	0.11	-0.75 to 0.96	0.80

[1] Each factor is adjusted for the other plus adjusted for severity of permanent childhood hearing impairment, maternal education level, non-verbal ability, and English as main language at home; late confirmation and no UNHS are reference category where appropriate.

YARC=York assessment of reading for comprehension; vs=versus; PCHI=Permanent childhood hearing impairment; UNHS=Universal newborn hearing screening.
